# 基于液相色谱-串联质谱的结肠癌血清氧固醇标志物筛选

**DOI:** 10.3724/SP.J.1123.2022.01001

**Published:** 2022-06-08

**Authors:** Zhanjun MA, Zhenguo LI, Huan WANG, Renjun WANG, Xiaofei HAN

**Affiliations:** 1.大连大学生命科学与技术学院, 辽宁省糖脂代谢研究重点实验室, 大连合成生物学重点实验室, 辽宁 大连 116622; 1. College of Life Science and Technology, Dalian University, Key Laboratory of Carbohydrate and Lipid Metabolism Research, Key Laboratory of Dalian Synthetic Biology, Dalian 116622, China; 2.大连医科大学附属第二医院, 辽宁 大连 116023; 2. The Second Hospital of Dalian Medical University, Dalian 116023, China

**Keywords:** 液相色谱-串联质谱, 结肠癌, 氧固醇, 代谢组学, liquid chromatography-tandem mass spectrometry (LC-MS/MS), colon cancer (CC), oxysterol, metabolomics

## Abstract

结肠癌(CC)是全球常见恶性肿瘤之一,发病率呈逐年上升趋势,目前没有有效的标志物用于疾病早期诊断和干预跟踪。胆固醇及其氧化衍生物氧固醇在众多恶性肿瘤发生发展中发挥关键作用。该研究采用液相色谱-串联质谱(LC-MS/MS)技术,对CC临床血清样本中胆固醇及相关10种氧固醇代谢物进行了定性定量分析,并采用偏最小二乘判别分析(PLS-DA)和正交偏最小二乘判别分析(OPLS-DA)进行多元统计分析,发现上述目标代谢物能够较好地区分CC组与健康对照组。为防止数据过拟合,该研究在PLS-DA模型各代谢物变量投影重要性(VIP)基础上,结合最优组分数及*K*-均值聚类结果,筛选得到3种代谢标志物。通过受试者操作特征曲线(ROC)的曲线下面积(AUC)分析,发现筛选得到的3种潜在标志物联合预测CC达到0.998,说明模型性能优良。GO(基因本体论)富集分析显示3种潜在标志物主要分布在内质网和包被囊泡上,参与胆固醇代谢、运输、低密度脂蛋白重塑等生物进程,发挥胆固醇运输活性和低密度脂蛋白颗粒受体结合的分子功能。KEGG(京都基因与基因组百科全书)通路分析显示3种潜在标志物富集于类固醇生物合成、PPAR(过氧化物酶体增殖物激活受体)信号通路及ABC(ATP结合盒)转运等通路上。该研究为寻找CC标志物及进一步阐明胆固醇及氧固醇在CC发病过程中的作用奠定了一定的基础。

## Abstract (en)

Colon cancer (CC) is one of the most common malignant tumors worldwide. As there are no effective biomarkers for the early diagnosis and intervention tracking, the incidence of CC is increasing every year. Cholesterol is an important component of cell membrane, and it has been shown to be associated with CC. Oxysterol is an oxidized derivative of cholesterol, which plays an important role in many malignant tumors. In this study, liquid chromatography-tandem mass spectrometry (LC-MS/MS) was used to determine serum cholesterol and ten oxysterol metabolites related to cholesterol in CC patients and healthy controls, and qualitative and quantitative analyses were carried out. Raw data were processed and analyzed using GraphPad Prism 8.3.0 and the MetaboAnalyst 5.0 platform (https://www.metaboanalyst.ca/MetaboAnalyst/ModuleView.xhtml). To perform the independent sample *t*-test, it was necessary to ensure that all the sample data followed a normal distribution; therefore, the normal distribution test was performed in advance. The Mann-Whitney U test, which is a nonparametric test, was adopted for samples without a normal distribution. For the processed data, we used the statistical analysis function module of the MetaboAnalyst 5.0 platform to perform partial least-square discriminant analysis (PLS-DA) and orthogonal partial least-square discriminant analysis (OPLS-DA). Both PLS-DA and OPLS-DA are supervised discriminant analysis methods. The OPLS-DA model is based on the PLS-DA model and eliminates variables that are unrelated to the experiment. In both models, the samples from the two groups were well separated by the score plot. In the PLS-DA model, the horizontal and vertical coordinates of the score plot represent the interpretation rates of the principal components of the model. The horizontal coordinates show the differences between groups, and the vertical coordinates show the differences within groups. In addition to the score plot in the PLS-DA model, another crucial factor is variable importance in the projection (VIP). When VIP>1, the compound makes an important contribution to the model and is also used as a criterion for screening differential metabolites. Based on 10-fold cross-validation (CV) of the PLS-DA model, the performance of the model was the best when the number of components was three. To avoid overfitting of the data, three metabolic markers were selected by using not only the VIP values of metabolites of the PLS-DA model, but also the optimal compositions and *K*-mean clusters. The three biomarkers were 4*β*-hydroxycholesterol (4*β*-OHC), cholestane-3*β*,5*α*,6*β*-triol (Triol), and cholesterol. A receiver operating characteristic (ROC) curve was constructed. The area under the curve (AUC) was generally between 0.5 and 1.0. In the case of AUC>0.5, the closer the AUC is to 1, the better is the performance of the model. In this study, the area under the ROC curve constructed jointly by the three metabolic markers was 0.998, indicating that their combined ability to predict CC was strong and that the diagnostic performance was excellent. In addition, to understand the role of the three metabolic markers in the pathogenesis of CC, the genes associated with the metabolic markers were identified using GeneCards (https://www.genecards.org/). Finally, 110 genes were identified. Gene ontology (GO) enrichment and Kyoto Encyclopedia of Genes and Genomes (KEGG) were used to analyze the biological processes, metabolic pathways, and possible roles in the body. GO enrichment showed that the three markers are mainly distributed in the endoplasmic reticulum lumen and coated vesicles, and they are mainly involved in biological processes such as cholesterol metabolism, transportation, and low-density lipoprotein particle remodeling. Their molecular functions are cholesterol transfer activity and low-density lipoprotein particle receptor binding. KEGG pathway analysis showed that biomarkers are enriched in steroid biosynthesis, PPAR (peroxisome proliferator-activated receptor) signaling pathways, and ABC (ATP-binding cassette) transport pathways. The results of this study are helpful to understand the role of cholesterol and oxysterol in the pathogenesis of CC and to elucidate the pathogenesis of CC.

结肠癌(colon cancer, CC)已经成为全球仅次于肺癌和乳腺癌的第三大恶性肿瘤,其发病不仅与年龄^[1]^、性别相关,还与遗传^[2]^、免疫和环境因素有关,如炎症^[3]^、饮酒^[4]^等,对生命和生活造成严重威胁。仅2018年就有大约200万病例被确诊为CC^[5]^。有研究证实,CC的发生发展与高脂低纤维饮食有着密切的联系^[6,7]^。我国经济的快速发展以及西方饮食文化的渗入,一定程度上提高了我国的CC患病率。结肠镜检查是CC诊断的“金标准”^[8]^,但它是侵入性的,可能引起患者的不适。基于血液检测的标记物如癌胚抗原和癌症抗原可以用来监测疾病以及对于治疗的反应,但是其敏感性和特异性较低,因此不太适合用作早期筛查和诊断^[9]^。粪便潜血试验(FOBT)和免疫组化试验(FIT)是基于粪便的DNA检测。其中FIT对CC有78%的敏感性和96%的特异性^[10]^,但是人们厌恶处理粪便样本,导致检测存在一定程度的挑战。因此发掘更加有效、能被广泛接受的标志物对于开展CC早期诊断和干预跟踪有重大意义。

胆固醇是细胞膜的重要组成部分,约占质膜脂质的20%,由于其在细胞中的特殊位置,对体内任何细胞新陈代谢可能都有未知的协调作用,同时在调节细胞膜流动性、细胞增殖等方面也发挥着关键作用^[11]^。一项研究表明,胆固醇是雌激素受体相关受体α(ERRα)的内源性配体(多种癌症的重要调节剂^[12]^)。一项与疾病相关的遗传因素研究证实了高胆固醇水平与CC风险之间的联系^[13]^。氧固醇是胆固醇的氧化衍生物,虽然氧固醇的生理功能尚不清楚,但在恶性肿瘤中起着重要作用^[14]^。对CC细胞系的研究表明,不同类型的氧固醇对细胞生命有不同的影响,有的能够促进细胞凋亡,有的能够抑制细胞增殖^[15]^。血清中的氧固醇含量非常低,大约是胆固醇含量的1/10^6^~1/1 ${0^{4}}^{}$ ^[16]^。如果浓度过高,会对细胞产生毒性作用,所以在健康机体中,过量的氧固醇被输送到肝脏,在那里转化为胆汁酸,重新进入人体循环^[17]^。因此,这些通路中涉及的基因或酶的突变或表达水平的变化极有可能诱发CC。

近年来代谢组学的发展极为迅速,结合代谢组学分析研究疾病的发生发展已经成为必不可少的一步。本文通过将液相色谱-串联质谱联用鉴定的代谢物导入代谢组学分析软件,筛选CC潜在标志物,期望能够为CC的临床诊断提供思路。

## 1 实验部分

### 1.1 仪器、试剂与材料

液相色谱仪配自动进样器、Surveyor MS Pump plus溶剂运输泵和Hypersil GOLD C18柱(150 mm×2.1 mm, 5 μm, Thermo Electron), TSQ Quantum Access Max三重四极杆质谱仪(Thermo Fisher Scientific,美国); G-560E涡旋振荡仪(Scientific Industries,美国); 5804R离心机(Eppendorf,德国); OA-SYS N-EVAP111氮吹仪(Organomation Associates,美国);恒温水浴锅(Fisher Scientific,美国); Milli-Q超纯水系统(Millipore,美国)。

氧固醇标准品(纯度均≥98%),包括24-羟基胆固醇(24-OHC)、7*α*-羟基胆固醇(7*α*-OHC)、7-酮基胆固醇(7-KC)、25-羟基胆固醇(25-OHC)、4*β*-羟基胆固醇(4*β*-OHC)、3*β*,5*α*,6*β*-三羟基胆固醇(Triol)、19-羟基胆固醇(19-OHC)(Toronto Research Chemicals,加拿大); 20*α*-羟基胆固醇(20*α*-OHC)、22*β*-羟基胆固醇(22*β*-OHC)、5*β*,6*β*-环氧胆固醇(5*β*,6*β*)(Sigma-Aldrich,美国); 5*α*,6*α*-环氧胆固醇(5*α*,6*α*)(Tokyo Chemical Industry,日本); 27-羟基胆固醇(27-OHC)(Tocris Bioscience,英国);胆固醇和氢氧化钾(百灵威,中国);甲酸、甲醇、乙腈均为色谱纯; 4-二甲氨基吡啶、吡啶甲酸、吡啶、三乙胺、2,6-二叔丁基-4-甲基苯酚(BHT)(Sigma-Aldrich,美国); 2-甲基-6-硝基苯酸酐(Bide Pharmatech,中国)。

### 1.2 样本采集与预处理

CC组(*n*=13)和健康对照组(HC, *n*=29)血液样本均来自大连医科大学附属第二医院,所有受试者的临床样本信息见表1。研究涉及的血液样本及程序均获得大连大学伦理委员会批准。血液样本室温下静置,凝固之后在3500 r/min下离心15 min,收集上清液即为血清,之后将得到的血清样本储存于-80 ℃,待LC-MS/MS分析。

**表1:** 血清样本的临床信息

Type	Gender (female/male)	Age	TC/(mmol/L)	TG/(mmol/L)	LDL-C/(mmol/L)	HDL-C/(mmol/L)
HC group (*n*=29)	14/15	38.79±11.84	4.48±0.76	1.21±0.36	2.34±0.63	1.41±0.32
CC group (*n*=13)	8/5	63.15±8.16	5.19±0.98	1.51±0.91	2.83±0.58	1.35±0.41
*P* value		<0.0001	0.0174	0.6048	0.0224	0.6137

HC: healthy control; CC: colon cancer; TC: total cholesterol; TG: triglyceride; LDL-C: low-density lipoprotein; HDL-C: high-density lipoprotein.

### 1.3 标准溶液的配制

准确称取适量的氧固醇标准品,配制成1.5 mg/mL储备液。取氧固醇储备液,用乙腈制成2.5 μg/mL混合标准中间液。19-羟基胆固醇用乙腈制备成2.5 μg/mL内标中间液和0.25 μg/mL内标工作液。用移液枪按一定比例取适量的混合标准中间液和内标中间液,分别制成5、10、20、50、100、200 ng/mL的混合标准工作液。

### 1.4 样品前处理

准确移取50 μL血清样品,加入10 μL内标、10 μL BHT乙醇溶液(0.02 mol/L)和150 μL氢氧化钾乙醇溶液(1 mol/L),之后在37 ℃水浴中反应1 h。再向其中加入蒸馏水和正己烷,4000 r/min下离心5 min,取上清液并用氮气吹干。吹干后加入衍生化试剂2-甲基-6硝基苯酸酐(1 mg)、4-二甲氨基吡啶(3 mg)、吡啶甲酸(8 mg)、吡啶(150 μL)、三乙胺(20 μL),置于37 ℃水浴反应2 h。为提取氧固醇衍生物,向反应后的混合物中加入1 mL正己烷,涡旋30 s混匀,4000 r/min下离心5 min,收集上清液并在氮气流下吹干。之后用100 μL乙腈复溶,取10 μL上样。

### 1.5 LC-MS/MS分析

Hypersil GOLD C18柱(150 mm×2.1 mm, 5 μm, Thermo Electron);色谱流动相组成:A(1%甲酸)-B(超纯水)-C(甲醇)-D(乙腈)。色谱梯度洗脱条件如下^[18]^: 0~15.0 min,5%A,15%B,10%C,70%D; 15.0~15.2 min,5%A,10%C,85%D; 15.2~20.2 min,10%C,90%D; 20.2~26.0 min,5%A,15%B,10%C,70%D。质谱参数设置:正离子模式检测,喷雾电压为3500 V,蒸发器温度保持在300 ℃,碰撞气体(氩气)压力固定在0.2 Pa。鞘气和辅助气均为氮气,各自压力保持在0.207 MPa和0.103 MPa。LC-MS/MS其他分析条件见文献^[18]^。

### 1.6 数据处理和统计分析

采用质谱仪配有的Xcalibur 2.1软件对数据进行采集与处理。最终将获得的数据导入MetaboAnalyst 5.0平台(https://www.metaboanalyst.ca/MetaboAnalyst/ModuleView.xhtml)进行数据标准化,并使用其中的统计学和生物标志物分析功能模块执行最小二乘判别分析(PLS-DA)、正交偏最小二乘判别分析(OPLS-DA)以及受试者操作特征曲线(ROC)绘制。最后为了解筛选到的代谢物在体内的相关代谢通路,阐明其在CC发病中的机制,我们使用GeneCards网站(https://www.genecards.org/)检索代谢物相关基因,进行后续的GO(基因本体论,gene ontology)功能注释和KEGG(京都基因与基因组百科全书,Kyoto Encyclopedia of Genes and Genomes)通路分析。富集分析的参数设置如下:最小重叠为3;截断*P*值<0.01;最小富集系数为1.5。

## 2 结果与讨论

### 2.1 生物标志物筛选

通过LC-MS/MS对CC患者和健康对照者血清中10种氧固醇进行了定性定量分析,并使用GraphPad Prism 8.3.0对两组间的代谢物进行显著性差异分析(*P*<0.05代表有显著性差异),结果见表2。

**表2:** CC组和HC组的血清胆固醇和氧固醇含量差异分析

Name	*P* value (HC vs CC)
20*α*-Hydroxycholesterol (20*α*-OHC)	0.9311
24-Hydroxycholesterol (24-OHC)+	0.3724
22*β*-hydroxycholesterol (22*β*-OHC)	
25-Hydroxycholesterol (25-OHC)	0.3862
7*α*-Hydroxycholesterol (7*α*-OHC)	0.9358
7-Ketocholesterol (7-KC)	0.5186
27-Hydroxycholesterol (27-OHC)	0.2379
Cholestane-3*β*,5*α*,6*β*-triol (Triol)	0.9358
Cholesterol-5*β*,6*β*-epoxide (5*β*,6*β*)	0.1590
Cholesterol-5*α*,6*α*-epoxide (5*α*,6*α*)	0.0506
4*β*-Hydroxycholesterol (4*β*-OHC)	0.0153
Cholesterol	<0.0001

通过MetaboAnalyst的统计学模块对两组样本的所有代谢物进行PLS-DA和OPLS-DA分析,可以看出两组样本均能够得到较好的分离(见图1)。对于PLS-DA模型,除了得分图以外,另一个重要的指标是变量投影重要性(VIP)。每一种代谢物的VIP值如图2a所示,代谢物VIP值越大,代表其对该模型的贡献度越大。依据每个组分对于模型的解释度,越靠后的组分解释度越低,因此我们选择了前8个组分进行10倍交叉验证(cross-validation, CV)。由于*Q*^2^表示模型的可预测性和准确性,我们选择了*Q*^2^来评估模型的性能。基于CV结果,我们发现当组分数量为3时(见图2b),模型的性能最好。为了防止数据过度拟合,我们还对代谢物进行了*K*-均值聚类(见表3),最终选择每一类别中VIP值较大的化合物作为后续ROC曲线的联合标志物组,它们分别是4*β*-OHC、Triol、胆固醇。

**图1:**
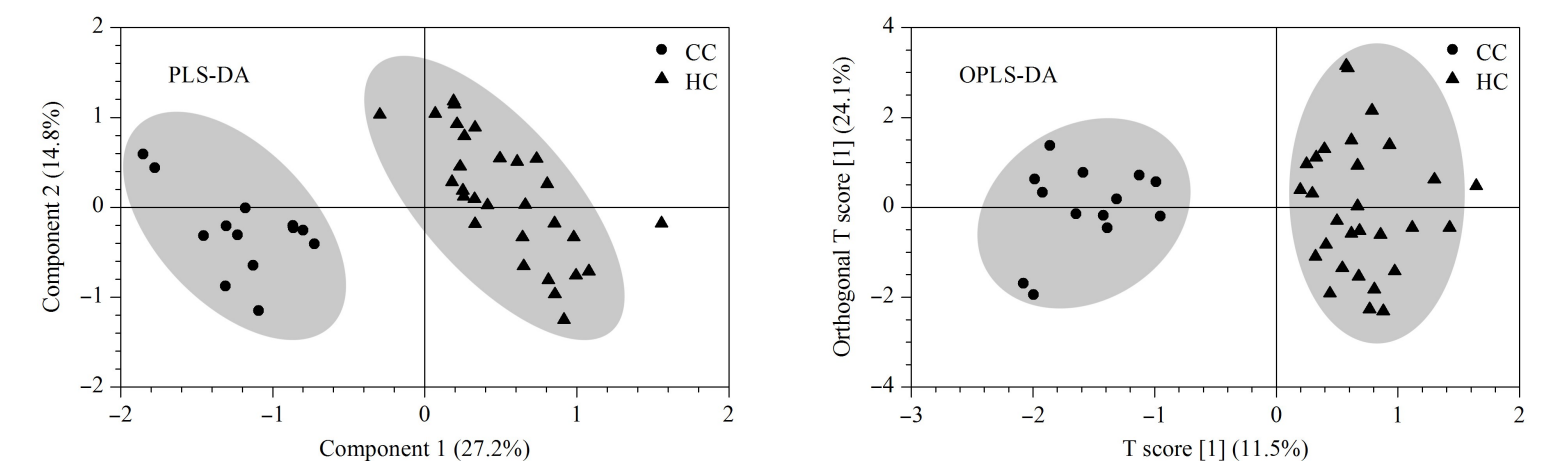
CC组和HC组的PLS-DA和OPLS-DA模型

**图2:**
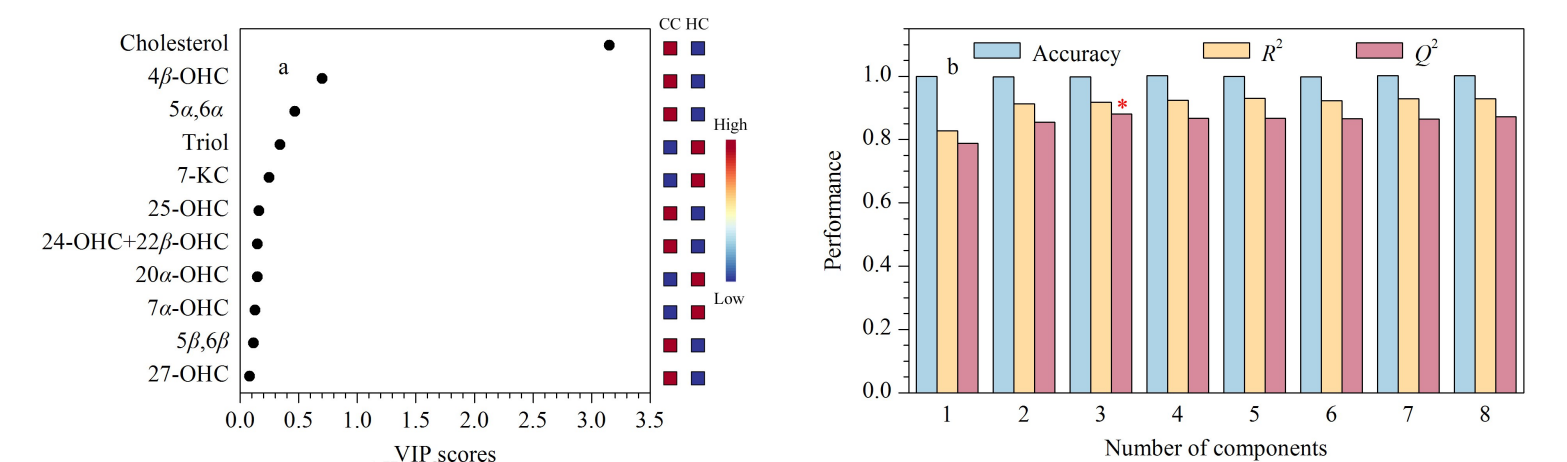
PLS-DA模型的(a)VIP值和(b)最优组分

**表3:** 代谢物的*K*-均值聚类

Name	*K*-mean cluster
20*α*-OHC	3
24-OHC+22*β*-OHC	3
25-OHC	2
7*α*-OHC	3
7-KC	3
27-OHC	3
Triol	3
5*β*,6*β*	2
5*α*,6*α*	2
4*β*-OHC	2
Cholesterol	1

### 2.2 ROC曲线验证

ROC曲线是一种二元分类器,能够用于分析阳性/阴性、有病/无病等情况。该研究利用筛选出的3种生物标志物联合绘制ROC曲线(见图3),曲线下面积(AUC)可以用来判断该分类器的性能。一般认为AUC值> 0.5时,其值越接近于1,说明此分类器的诊断效果越好。图3的AUC为0.998,表明联合3种代谢物预测CC的效果较好。

**图3:**
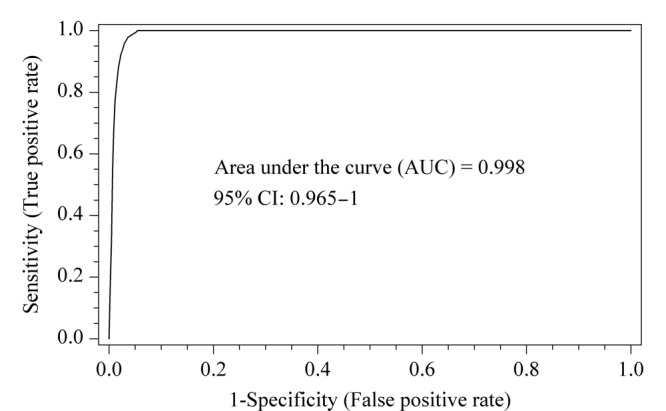
联合4*β*-OHC、Triol、胆固醇绘制ROC曲线

### 2.3 GO富集和KEGG通路分析

我们使用GeneCards网站检索了4*β*-OHC、Triol和胆固醇的相关基因。由于与胆固醇相关的基因数目很多,我们选择了前100个基因进行后续分析。我们利用Metascape (https://metascape.org/gp/index.html#/main/step1)和微生信在线软件(http://www.bioinformatics.com.cn/)识别到了110个基因中的103个人源基因,并对其进行了GO注释和KEGG通路分析。GO分析结果表明,代谢物相关基因主要参与的生物过程是胆固醇代谢、运输、调节脂蛋白沉积和重塑。它们所执行的分子功能是胆固醇运输活性和低密度脂蛋白颗粒受体结合,定位于内质网和包被囊泡(见图4a)。通路分析表明,最显著富集的途径是类固醇生物合成、PPAR(过氧化物酶体增殖物激活受体)信号通路和ABC(ATP结合盒)转运(见图4b)。

**图4:**
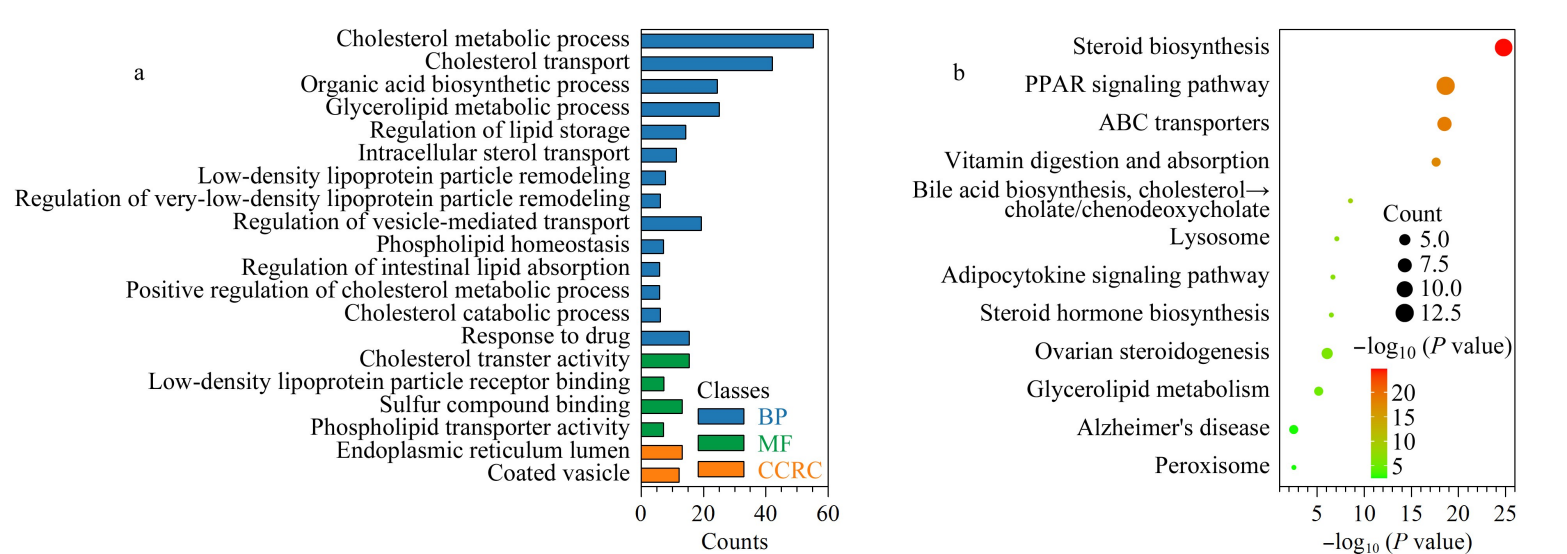
4*β*-OHC、Triol和胆固醇相关基因的(a)GO富集和(b)KEGG通路

## 3 结论

通过LC-MS/MS对CC患者血清氧固醇的定性、定量,结合代谢组学多维度分析,筛选出4*β*-OHC、Triol、胆固醇作为CC的潜在生物标志物,且这3种代谢物建立的分类器性能良好,为临床诊断和治疗疾病提供了可能。此外,对标志物的通路分析揭示了其参与的相关生物学进程,有助于更好地阐明CC的发病机理。
